# Duration of SARS-CoV-2 shedding: A systematic review

**DOI:** 10.7189/jogh.14.05005

**Published:** 2024-03-29

**Authors:** Anouk M Oordt-Speets, Julia R Spinardi, Carlos F Mendoza, Jingyan Yang, Graciela del Carmen Morales, Moe H Kyaw

**Affiliations:** 1 Epi-C (Epidemiology-Consultancy), the Netherlands; 2 Vaccine Medical Affairs, Emerging Markets, Pfizer Inc., Itapevi, Brazil; 3 Patient Health and Impact, Pfizer Inc., Mexico City, Mexico; 4 Global Value and Access, Pfizer Inc., New York, USA; 5 Vaccine Medical Affairs, Emerging Markets, Pfizer Inc., New York, USA; 6 Vaccine Scientific Affairs, Emerging Markets, Pfizer Inc., New York, USA

## Abstract

**Background:**

Positive viral severe acute respiratory syndrome coronavirus 2 (SARS-CoV-2) cultures indicate shedding of infectious virus and corresponding transmission risk of coronavirus disease 2019 (COVID-19). The research question of this systematic review was: Is there a discernible pattern in the timing of SARS-CoV-2 virus isolation, and what is the proportion of positive and negative results for isolation of SARS-CoV-2 virus with viral culture relative to the onset of clinical symptoms or the day of diagnosis, as indicated by longitudinal studies?

**Methods:**

We systematically searched PubMed and Embase from inception to 16 February 2023 for English-language studies with serial viral culture testing within symptomatic or asymptomatic SARS-CoV-2 infected persons during the post-vaccination period. Outcomes of interest were the daily culture status per study and the overall daily culture positivity rate of SARS-CoV-2. We critically appraised the selected studies using the Newcastle-Ottawa quality assessment scale.

**Results:**

We included 14 viral shedding studies in this systematic review. Positive viral SARS-CoV-2 cultures were detected in samples ranging from 4 days before to 18 days after symptom onset. The daily culture SARS-CoV-2 positivity rate since symptom onset or diagnosis showed a steep decline between day 5 and 9, starting with a peak ranging from 44% to 50% on days −1 to 5, decreasing to 28% on day 7 and 11% on day 9, and finally ranging between 0% and 8% on days 10–17.

**Conclusions:**

Viral shedding peaked within 5 days since symptom onset or diagnosis and the culture positivity rate rapidly declined hereafter. This systematic review provides an overview of current evidence on the daily SARS-CoV-2 culture positivity rates during the post-vaccination period. These findings could be used to estimate the effectiveness of public health control measures, including treatment and preventive strategies, to reduce the spread of COVID-19.

An important characteristic of an infectious disease is that infected persons can become the sources of exposure; the exact duration of the time when an infected person can transmit the infectious agent to others – known as the infectious period – is unique to each infection [1]. Understanding the duration of shedding of the novel severe acute respiratory syndrome coronavirus 2 (SARS-CoV-2) and its impact on the transmission of coronavirus disease 2019 (COVID-19) is crucial for designing public health control measures such as quarantine, isolation, and contact tracing, as well as discerning the potential impact of antiviral treatments or vaccination [2].

Real-time reverse transcriptase polymerase chain reaction (RT-PCR) tests are the gold standard for SARS-CoV-2 diagnosis [3,4]. However, in infected persons, RT-PCR can remain positive for weeks or months following the resolution of clinical illness, without allowing for differentiation between the shedding of viable and potentially infective viruses and non-infectious viral fragments [3,4]. Although this is not believed to represent ongoing viral replication in most cases [3], characteristics such as disease severity and immunosuppressive status have been shown to be related to possible prolonged viable SARS-CoV-2 shedding [4]. The recovery of SARS-CoV-2 in viral culture is currently the sole approach for determining the presence of the infectious virus and continued transmission risk [3,4]. Due to biosafety level 3 requirements, the use of viral culture is not feasible routinely and is usually restricted to research only [3,4]. For example, the World Health Organization (WHO) and Centers for Disease Control and Prevention (CDC) guidelines on the isolation period for COVID-19 patients were based on serial viral culture studies and had changed during the pandemic [5,6]. The research question of this systematic review was: Is there a discernible pattern in the timing of SARS-CoV-2 virus isolation, and what is the proportion of positive and negative results for isolation of SARS-CoV-2 virus with viral culture relative to the onset of clinical symptoms or the day of diagnosis, as indicated by longitudinal studies?

## METHODS

We conducted this systematic review following a pre-designed protocol, which is available from the corresponding author upon request. We systematically searched PubMed and Embase from inception to 16 February 2023 for English-language articles. The search strings used in this process were composed of keywords related to SARS-CoV-2-infected persons and the outcome of virus shedding as isolated with viral culture (Table S1 in the **Online Supplementary Document**). Outcomes of interest were the daily culture status per study (i.e. per day rated as positive, when at least one positive viral culture was found, or negative) and the overall daily culture positivity rate of SARS-CoV-2. For the studies which reported the number of positive and negative cultures per day, we calculated an overall daily SARS-CoV-2 culture positivity.

We selected relevant articles based on pre-specified eligibility criteria, following a two-step approach of title/abstract and full-text screening. One researcher (AMO) screened the titles/abstracts in close collaboration with a second researcher (MHK), resolving disagreements through discussion or (if they persisted) by screening the full text of the article in question. The full-text selection was likewise done by two researchers. Disagreements were resolved through discussion until a consensus was reached; otherwise, a third researcher (JRS) was consulted to resolve any issues. We additionally screened the reference lists of the included studies and relevant reviews; however, we found no eligible studies at this stage. We used the Rayyan software [7] in conducting the screening process.

We included studies with serial viral culture testing within symptomatic or asymptomatic SARS-CoV-2 infected persons during the post-vaccination period, regardless of the vaccination coverage. Meanwhile, we excluded studies: without serial samples within individuals; conducted during the pre-vaccination period (before December 2020) or without a reported study period; with an unvaccinated population only; of irrelevant publication type (e.g. comment, reviews); and those published in several articles, where we included the one with most complete data. We initially selected preprints as well, but only used them to search for possible peer-reviewed, published versions.

One researcher (AMO) extracted the characteristics and relevant data from the included studies into Microsoft Excel 2021 MSO, version 2402 (Microsoft Inc., Redmond, Washington, USA), with a second researcher (MHK) reviewing the extracted data and discussing discrepancies. In cases of incomplete or unclear data, we reached out to authors to provide the missing information (five authors were contacted; three responded).

One researcher (AMO) then critically appraised the studies with the Newcastle-Ottawa quality assessment scale for cohort studies [8]; a second researcher (MHK) reviewed the evaluation and discussed disagreements with the first researcher.

## RESULTS

**Figure 1:**
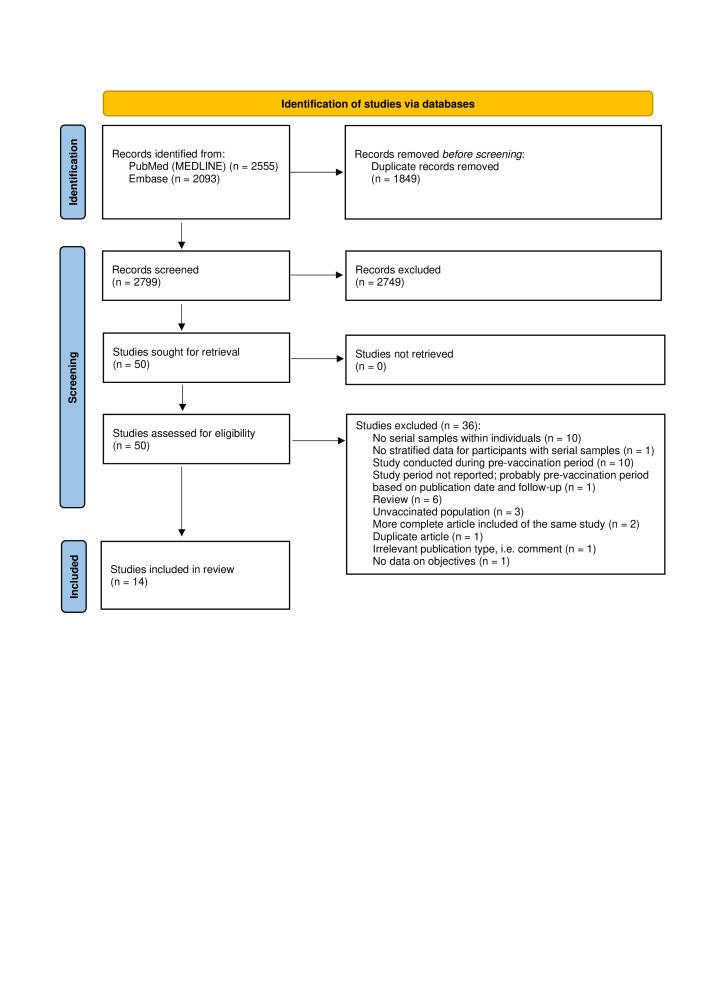
PRISMA flow diagram.

We screened the title/abstracts of 2799 unique records retrieved from PubMed and Embase, and the full texts of 50 studies remaining after this stage. We selected 14 prospective cohort studies for inclusion in this systematic review (**Figure 1**; Table S2 in the **Online Supplementary Document**) [9–22].

### Study characteristics

**Figure 2:**
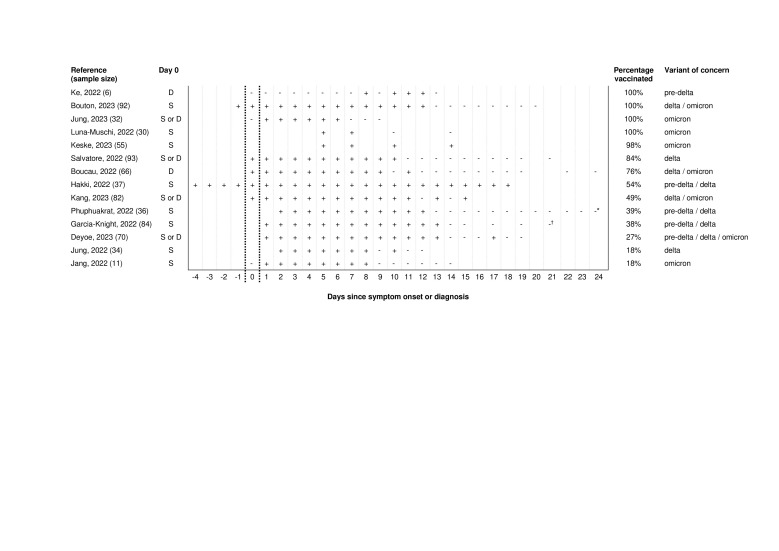
Daily SARS-CoV-2 culture status since symptom onset or diagnosis (n = 14 studies). S: day 0 defined as first day of symptom onset (i.e. symptomatic cases); D: day 0 defined as first positive diagnostic SARS-CoV-2 test in symptomatic and asymptomatic cases; S or D: day 0 defined as first day of symptom onset (i.e. symptomatic cases) or diagnosis (i.e. asymptomatic cases). *Culture negative up to day 32. †Plus a final negative culture on day 28 (not shown in this figure).

Most studies were conducted in Northern America (43%) or Asia (36%) and in adults (86%), with sample sizes ranging from 6 to 93. Different SARS-CoV-2 infected populations were studied: non-hospitalised persons (21%), health care workers (21%), university students/employees (14%), prisoners (7%), primary cases in households (7%), isolated patients (21%), and hospitalised patients (7%). They were conducted in periods of the pre-Delta, Delta, and Omicron SARS-CoV-2 variants of concern. The vaccination coverage with primary series or first booster in the study populations ranged from 18% to 100% (**Figure 2** and **Table 1**; Table S3 in the **Online Supplementary Document**).

**Table 1:** Overview study characteristics of the included studies

Study characteristics	n (%)
Number of studies	14
Study design – prospective cohort study	14 (100%)
Region	
*Northern America*	6 (42.9%)
*Southern America*	1 (7.1%)
*Europe*	1 (7.1%)
*Middle East*	1 (7.1%)
*Asia*	5 (35.7%)
Setting/population	
*Non-hospitalised SARS-CoV-2-positive persons*	3 (21.4%)
*Non-hospitalised SARS-CoV-2-positive health care workers*	3 (21.4%)
*SARS-CoV-2 positive university students and employees*	2 (14.3%)
*SARS-CoV-2 positive prisoners*	1 (7.1%)
*Primary cases in households*	1 (7.1%)
*Isolated or mandatorily hospitalised non-critically ill COVID-19 patients*	3 (21.4%)
*Hospitalised patients with mild to moderate COVID-19*	1 (7.1%)
Sample size, range	6-93
Male, range	22–100%
Age	
*Mean age, range*	22–42 y
*Median age, range*	22-67 y
Definition day zero for the viral culture serial sampling	
*Symptom onset*	8 (57.1%)*
*Diagnosis*	2 (14.3%)
*Symptom onset or diagnosis*	4 (28.6%)
SARS-CoV-2 variant of concern	
*Pre-Delta*	1 (7.1%)
*Pre-Delta/Delta*	3 (21.4%)
*Delta*	2 (14.3%)
*Pre-Delta/Delta/Omicron*	1 (7.1%)
*Delta/Omicron*	3 (21.4%)
*Omicron*	4 (28.6%)
Vaccination coverage in the study population, range	18–100%
COVID-19 vaccine	
*mRNA vaccine*	1 (7.1%)
*mRNA or vector vaccine*	4 (28.6%)
*mRNA or inactivated vaccine*	1 (7.1%)
*mRNA or vector or inactivated vaccine*	2 (14.3%)
*Vector or inactivated vaccine*	1 (7.1%)
*Not reported*	5 (35.7%)
Risk of bias assessment	
*Low risk of bias*	0 (0%)
*Moderate risk of bias*	12 (85.7%)
*High risk of bias*	2 (14.3%)

*One study reported data separately for ‘since symptom onset’ and ‘since diagnosis.’ To avoid duplicate data, we extracted only the ‘since symptom onset.’

Day zero in the serial culture sampling was not defined uniformly in the studies: symptom onset (i.e. symptomatic cases) in eight studies; symptom onset or diagnosis (i.e. asymptomatic cases) in four studies; and diagnosis (i.e. first positive PCR test in symptomatic and asymptomatic cases) in two studies. The studies had a moderate (86%) or high (14%) risk of bias based on the Newcastle-Ottawa scale (Table S4 in the **Online Supplementary Document**).

### Daily SARS-CoV-2 culture status

The daily SARS-CoV-2 culture status of each included study shows per day when at least one positive viral culture was found (**Figure 2**). A positive viral culture was detected in samples ranging from 4 days before symptom onset to 18 days after symptom onset. Four of the 14 studies (29%) had detected positive viral culture 14 days after symptom onset or diagnosis.

### Daily SARS-CoV-2 culture positivity rates

**Figure 3:**
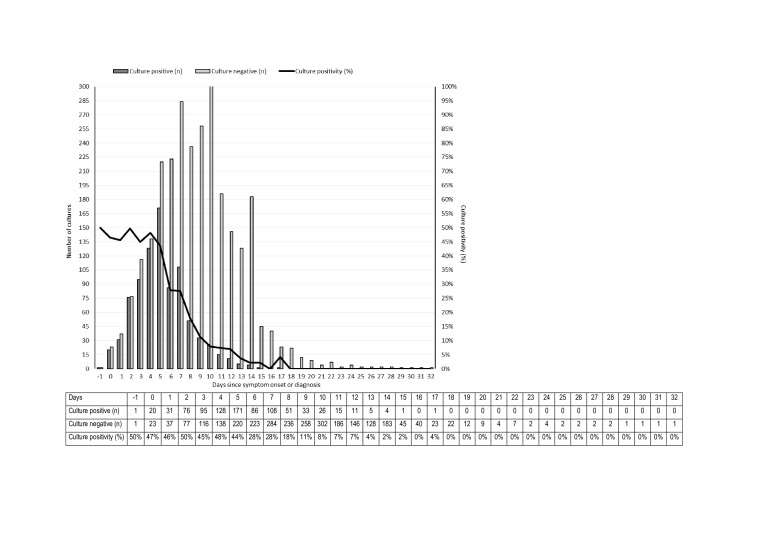
Daily SARS-CoV-2 culture positivity since symptom onset or diagnosis (n = 11 studies). Three studies were not included in this figure, because the data was not complete to calculate culture positivity: Hakki et al. (2022) [13], Jang et al. (2022) [14], Jung et al. (2022) [16].

We were able to calculate daily culture positivity rates for 11 of the 14 studies (**Figure 3**) [9–12,15,17–22]. We found the daily culture positivity rate of SARS-CoV-2 to be up to 50% since symptom onset or diagnosis in the combined data of all cases. The culture positivity was 44% to 50% on days −1 to 5, followed by a steep decline to 28% at day 7 and 11% at day 9. Meanwhile, the daily percentages ranged between 0% and 8% for the days 10 to 17; viable virus in cell culture was found in none of the included studies beyond day 18 since symptom onset or diagnosis (**Figure 3**).

### Stratified groups

The number of studies was too small and had insufficient statistical power to show clear trends of daily SARS-CoV-2 culture status or culture positivity for stratified groups, such as vaccinated vs unvaccinated persons; different SARS-CoV-2 variants of concern; symptomatic vs asymptomatic SARS-CoV-2 infected persons; and time since symptom onset vs time since diagnosis (**Figure 2**).

## DISCUSSION

Among the included studies, positive viral SARS-CoV-2 cultures were mostly detected in serial samples within 14 days after symptom onset or diagnosis, while the daily culture positivity rate of SARS-CoV-2 showed a steep decline between days 5 and 9. These data on positive viral SARS-CoV-2 cultures help determine the time window of shedding of infectious virus and the corresponding transmission risk of COVID-19. We observed that SARS-CoV-2 viral shedding in the included studies peaked on days −1 to 5 since symptom onset or diagnosis; therefore, the implementation of public health control measures (such as quarantine and isolation) and the administration of antiviral treatments could offer the most potential benefits in this period. This is in line with the recommendations by the WHO and CDC, which during the COVID-19 period shortened the recommended isolation and quarantine period of five days after symptom onset or positive test [5,6]. Our findings also support the current CDC recommendation of five days of isolation followed by five days of mask-wearing for those with COVID-19 in the general population [23], since some patients with COVID-19 are possibly still infectious beyond five days since symptom onset or diagnosis (i.e. decreasing from 28% at days 6 and 7, to 11% at day 9, and 0% on day 18; **Figure 3**). Based on data from past infectious disease outbreaks, studies have suggested that isolation is the most important prevention and control measure for limiting the spread of infectious diseases [10]. Therefore, strict adherence to current isolation recommendations will likely reduce the impact of COVID-19 on the health care systems.

The SARS-CoV-2 culture positivity rate among the included studies was not more than 50%. Notably, the inclusion of symptomatic as well as asymptomatic cases in 6 of the 11 studies will have lowered the maximum percentage of SARS-CoV-2 culture positivity, compared to including symptomatic cases only. When we restricted to studies with symptomatic cases only, the upper range of the SARS-CoV-2 culture positivity rate was 57%. However, the real-world situation presents a mixture of symptomatic and asymptomatic SARS-CoV-2 cases. Furthermore, SARS-CoV-2 culture positivity and duration of virus shedding will be further influenced by other factors impacting the ability to clear the virus, such as disease severity; immunocompetence and comorbidities; vaccine impact or natural immunity of COVID-19; and the characteristics of pathogens such as the various SARS-CoV-2 variant of concern [4]. In this systematic review, we only looked at the post-vaccination period, as it encompassed the current COVID-19 circumstances, whereby much of the population has derived some form of immunity from previous infection and/or COVID-19 vaccination. Post-vaccination data will be more relevant for future control measures than outdated pre-vaccination data.

The studies included in this systematic review used viral culture for detecting infectious SARS-CoV-2. Recovery of SARS-CoV-2 in viral culture has served as the reference standard for detecting infectious viruses throughout the COVID-19 pandemic and is currently the only approach for determining the presence of the infectious virus [3,4]. The procedure of virus isolation is a reliable tool and is conducted by specially trained personnel in laboratories with advanced infrastructure, therefore providing more comparable results across studies than, for example, approaches using RT-PCR tests [3,4]. Though viral culture is considered the reference standard, there are some limitations. Specifically, detection of viable virus particles is highly influenced by the quality of the sample, which emphasises suitable storage conditions; cell lines used for isolation can show variability between laboratories; and consumables or additives used during cell culture could potentially also impact virus isolation success [4].

This systematic review has several limitations. We were able to include a limited number of studies with serial SARS-CoV-2 culture sampling, with small sample sizes and an even smaller number of serial samples per day for estimating the daily culture positivity rates. A part of the studies had incomplete data (e.g. missing data on negative or total cultures) or presented data in figures only without sufficient details to allow for extraction. However, we contacted the corresponding authors to address this issue. Furthermore, the data were heterogeneous (e.g. specimen collection, definition of day zero, populations, SARS-CoV-2 variant of concern, vaccination coverage), and the limited numbers of daily samples hampered comparing stratified results and identifying risk factors of prolonged SARS-CoV-2 virus shedding. Well-designed future studies with larger sample sizes and daily sample collection for viral culture, before and after symptom onset or diagnosis and across various age groups and different populations, will provide further understanding of the duration and determinants of SARS-CoV-2 virus shedding.

## CONCLUSIONS

In this systematic review, we found that the period of viral SARS-CoV-2 shedding occurred within five days after the symptom onset or diagnosis and that the culture positivity rate rapidly declined after this period. Our findings provide an overview of the current evidence on the daily SARS-CoV-2 culture positivity rates during the post-vaccination period, and could therefore be used to estimate the effectiveness of public health control measures, including treatment approaches and preventive strategies, aimed at reducing the spread of COVID-19.

## Additional material


Online Supplementary Document
