# Supplementary material for: Duration of SARS-CoV-2 shedding: A systematic review
Source: J Glob Health. 2024 Mar 29;14:05005. doi: 10.7189/jogh.14.05005 (PMC10978056; doi:10.7189/jogh.14.05005)
Supplement: Online Supplementary Document [file jogh-14-05005-s001.pdf]

Online Supplementary Document

Table S1. Search strategy

	PubMed (MEDLINE)	Embase
**Population**	"COVID-19"[Mesh] OR COVID-19[tiab] OR COVID19[tiab] OR 2019 novel coronavirus[tiab] OR 2019-nCoV[tiab] OR 2019 nCoV[tiab] OR coronavirus disease 2019[tiab] OR coronavirus disease-19[tiab] OR severe acute respiratory syndrome coronavirus 2[tiab] OR SARS coronavirus 2[tiab] OR SARS-CoV-2[tiab]	'coronavirus disease 2019'/exp OR 'covid 19':ti,ab OR covid19:ti,ab OR '2019 novel coronavirus':ti,ab OR '2019 ncov':ti,ab OR 'coronavirus disease 2019':ti,ab OR 'coronavirus disease-19':ti,ab OR 'severe acute respiratory syndrome coronavirus 2':ti,ab OR 'sars coronavirus 2':ti,ab OR 'sars cov 2':ti,ab
**Exposure**	No search string	No search string
**Comparator**	No search string	No search string
**Outcomes**	("Infectious Disease Incubation Period"[Mesh] OR incubation[tiab] OR infectious period[tiab] OR infectiousness[tiab] OR infectivity[tiab] OR transmissibility[tiab] OR communicability[tiab] OR "Virus Shedding"[Mesh] OR shed*[tiab] OR viral kinetics[tiab] OR viral load kinetics[tiab] OR viral dynamics[tiab] OR viral load dynamics[tiab] OR transmission kinetics[tiab] OR transmission dynamics[tiab] OR temporal[tiab] OR serial[tiab] OR repeated[tiab] OR day[tiab] OR days[tiab]) AND (cultur*[tiab] OR virus isolation[tiab] OR viral isolation[tiab] OR replication*[tiab] OR infectious virus[tiab] OR viable[tiab] OR viability[tiab])	('incubation time'/exp OR incubation:ti,ab OR 'infectious period':ti,ab OR infectiousness:ti,ab OR infectivity:ti,ab OR transmissibility:ti,ab OR communicability:ti,ab OR 'virus shedding'/exp OR shed*:ti,ab OR 'viral kinetics':ti,ab OR 'viral load kinetics':ti,ab OR 'viral dynamics':ti,ab OR 'viral load dynamics':ti,ab OR 'transmission kinetics':ti,ab OR 'transmission dynamics':ti,ab OR temporal:ti,ab OR serial:ti,ab OR repeated:ti,ab OR day:ti,ab OR days:ti,ab) AND (cultur*:ti,ab OR 'virus isolation':ti,ab OR 'viral isolation':ti,ab OR replication*:ti,ab OR 'infectious virus':ti,ab OR viable:ti,ab OR viability:ti,ab)
**Limits**	Language: English	Language: English
	No animal studies: NOT (Animals[Mesh] NOT (Humans[Mesh] AND Animals[Mesh]))	No animal studies: NOT ('animal'/exp OR 'nonhuman'/exp NOT ('animal'/exp OR 'nonhuman'/exp AND 'human'/exp))
**Search date**	16 February 2023	16 February 2023

Table S2. Primary reasons for exclusion by each excluded study at full-text review

Reference	Reason for exclusion
Antar AAR, Yu T, Pisanic N, Azamfirei R, Tornheim JA, Brown DM, et al. Delayed Rise of Oral Fluid Antibodies, Elevated BMI, and Absence of Early Fever Correlate with Longer Time to SARS-CoV-2 RNA Clearance in a Longitudinally Sampled Cohort of COVID-19 Outpatients. Open Forum Infectious Diseases. 2021;8(6).	Study conducted during pre-vaccination period
Bae S, Kim JY, Lim SY, Park H, Cha HH, Kwon JS, et al. Dynamics of viral shedding and symptoms in patients with asymptomatic or mild COVID-19. Viruses. 2021;13(11).	Unvaccinated population
Basile K, McPhie K, Carter I, Alderson S, Rahman H, Donovan L, et al. Cell-based Culture Informs Infectivity and Safe De-Isolation Assessments in Patients with Coronavirus Disease 2019. Clin Infect Dis. 2021;73(9):e2952-e9.	No stratified data for participants with serial samples
Bhat V, Chavan P, Khattry N, Gupta S. Dynamics of viral RNA load, virus culture, seroconversion & infectivity in COVID-19 patients: Implications on isolation policy. Indian Journal of Medical Research. 2021;153(5):585-90.	Review
Bonenfant G, Deyoe JE, Wong T, Grijalva CG, Cui D, Talbot HK, et al. Surveillance and Correlation of Severe Acute Respiratory Syndrome Coronavirus 2 Viral RNA, Antigen, Virus Isolation, and Self-Reported Symptoms in a Longitudinal Study With Daily Sampling. Clinical Infectious Diseases. 2022;75(10):1698-705.	Study conducted during pre-vaccination period
Bravo MS, Nicolás D, Berengua C, Fernandez M, Hurtado JC, Tortajada M, et al. Severe Acute Respiratory Syndrome Coronavirus 2 Normalized Viral Loads and Subgenomic RNA Detection as Tools for Improving Clinical Decision Making and Work Reincorporation. Journal of Infectious Diseases. 2021;224(8):1325-32.	No data on objectives
Cevik M, Kalil AC. Omicron variant: assessing the duration of viral shedding and its implications. Clin Microbiol Infect. 2023;29(2):133-4.	Review
Cosimi LA, Kelly C, Esposito S, Seitz S, Turcinovic J, Connor JH, et al. Duration of symptoms and association with positive home rapid antigen test results after infection with SARS-CoV-2. JAMA Network Open. 2022;5(8):e2225331-e.	No serial samples within individuals
Drain PK, Dalmat RR, Hao L, Bemer MJ, Budiawan E, Morton JF, et al. Duration of viral infectiousness and correlation with symptoms and diagnostic testing in non-hospitalized adults during acute SARS-CoV-2 infection: A longitudinal cohort study. Journal of Clinical Virology. 2023:105420.	Unvaccinated population
Gniazdowski V, Paul Morris C, Wohl S, Mehoke T, Ramakrishnan S, Thielen P, et al. Repeated Coronavirus Disease 2019 Molecular Testing: Correlation of Severe Acute Respiratory Syndrome Coronavirus 2 Culture with Molecular Assays and Cycle Thresholds. Clinical Infectious Diseases. 2021;73(4):E860-E9.	Study conducted during pre-vaccination period
Kang SW, Park H, Kim JY, Park S, Lim SY, Lee S, et al. Clinical scoring system to predict viable viral shedding in patients with COVID-19. Journal of Clinical Virology. 2022;157.	Duplicate data
Ke R, Martinez PP, Smith RL, Gibson LL, Mirza A, Conte M, et al. Daily longitudinal sampling of SARS-CoV-2 infection reveals substantial heterogeneity in infectiousness. Nature Microbiology. 2022;7(5):640-52.	Study conducted during pre-vaccination period
Killerby ME, Ata Ur Rasheed M, Tamin A, Harcourt JL, Abedi GR, Lu X, et al. Shedding of Culturable Virus, Seroconversion, and 6-Month Follow-up Antibody Responses in the First 14 Confirmed Cases of Coronavirus Disease 2019 in the United States. Journal of Infectious Diseases. 2021;224(5):771-6.	Study conducted during pre-vaccination period
Kim MC, Cui C, Shin KR, Bae JY, Kweon OJ, Lee MK, et al. Duration of culturable SARS-CoV-2 in hospitalized patients with Covid-19. New England Journal of Medicine. 2021;384(7):671-3.	Study conducted during pre-vaccination period

Kojima N, Mores C, Farsai N, Klausner J. Duration of SARS-CoV-2 viral culture positivity among different specimen types. Clin Microbiol Infect. 2021;27(10):1540-1.	No serial samples within individuals
Laferl H, Kelani H, Seitz T, Holzer B, Zimpernik I, Steinrigl A, et al. An approach to lifting self-isolation for health care workers with prolonged shedding of SARS-CoV-2 RNA. Infection. 2021;49(1):95-101.	Study conducted during pre-vaccination period
Letizia AG, Smith DR, Ge Y, Ramos I, Sealfon RSG, Goforth C, et al. Viable virus shedding during SARS-CoV-2 reinfection. The Lancet Respiratory Medicine. 2021;9(7):e56-e7.	No serial samples within individuals
Lewis NM, Duca LM, Marcenac P, Dietrich EA, Gregory CJ, Fields VL, et al. Characteristics and timing of initial virus shedding in severe acute respiratory syndrome coronavirus 2, Utah, USA. Emerging Infectious Diseases. 2021;27(2):352-9.	Study conducted during pre-vaccination period
Lohse ZM, Shapiro JJ, Lednicky JA, Cash MN, Jun I, Mavian CN, et al. Persistence of Severe Acute Respiratory Syndrome Coronavirus 2 Omicron Variant in Children and Utility of Rapid Antigen Testing as an Indicator of Culturable Virus. Clinical infectious diseases : an official publication of the Infectious Diseases Society of America. 2023;76(3):e491-e4.	No serial samples within individuals
McCormick DW, Hagan LM, Salvatore PP, Magleby R, Lee C, Sleweon S, et al. SARS-CoV-2 viral shedding in vaccinated and unvaccinated persons: A case series. Vaccine. 2022.	More complete article included of the same study
Mollan KR, Eron JJ, Krajewski TJ, Painter W, Duke ER, Morse CG, et al. Infectious Severe Acute Respiratory Syndrome Coronavirus 2 (SARS-CoV-2) Virus in Symptomatic Coronavirus Disease 2019 (COVID-19) Outpatients: Host, Disease, and Viral Correlates. Clinical Infectious Diseases. 2022;75(1):E1028-E36.	No serial samples within individuals
Murata T, Sakurai A, Suzuki M, Komoto S, Ide T, Ishihara T, et al. Shedding of Viable Virus in Asymptomatic SARS-CoV-2 Carriers. mSphere. 2021;6(3):1-7.	Study conducted during pre-vaccination period
Ouoba S, Okimoto M, Nagashima S, Kitahara Y, Miwata K, Ko K, et al. Sequential dynamics of virological and serological changes in the serum of SARS-CoV-2 infected patients. Journal of Medical Virology. 2022;94(4):1734-7.	No serial samples within individuals
Owusu D, Pomeroy MA, Lewis NM, Wadhwa A, Yousaf AR, Whitaker B, et al. Persistent SARS-CoV-2 RNA Shedding Without Evidence of Infectiousness: A Cohort Study of Individuals With COVID-19. J Infect Dis. 2021;224(8):1362-71.	No serial samples within individuals
Park S, Lim SY, Kim JY, Park H, Lim JS, Bae S, et al. Clinical and Virological Characteristics of Severe Acute Respiratory Syndrome Coronavirus 2 (SARS-CoV-2) B.1.617.2 (Delta) Variant: A Prospective Cohort Study. Clinical Infectious Diseases. 2022;75(1):E27-E34.	No serial samples within individuals
Puhach O, Adea K, Hulo N, Sattonnet P, Genecand C, Iten A, et al. Infectious viral load in unvaccinated and vaccinated individuals infected with ancestral, Delta or Omicron SARS-CoV-2. Nature Medicine. 2022;28(7):1491-500.	No serial samples within individuals
Puhach O, Meyer B, Eckerle I. SARS-CoV-2 viral load and shedding kinetics. Nat Rev Microbiol. 2023;21(3):147-61.	Review
Qutub M, Aldabbagh Y, Mehdawi F, Alraddadi A, Alhomsy M, Alnahdi A, et al. Duration of viable SARS-CoV-2 shedding from respiratory tract in different human hosts and its impact on isolation discontinuation polices revision; a narrative review. Clinical Infection in Practice. 2022;13.	Review
Rahmani A, Dini G, Leso V, Montecucco A, Kusznir Vitturi B, Iavicoli I, et al. Duration of SARS-CoV-2 shedding and infectivity in the working age population: a systematic review and meta-analysis. La Medicina del lavoro. 2022;113(2):e2022014.	Review
Rhee C, Kanjilal S, Baker M, Klompas M. Duration of Severe Acute Respiratory Syndrome Coronavirus 2 (SARS-CoV-2) Infectivity: When Is It Safe to Discontinue Isolation? Clinical Infectious Diseases. 2021;72(8):1467-74.	Review
Romano CM, Felix AC, Pannuti CS. Viable SARS-CoV-2 Shedding. The New England journal of medicine. 2021;384(17):1677.	Irrelevant publication type (i.e. comment)

Sejdic A, Frische A, Jørgensen CS, Rasmussen LD, Trebbien R, Dungu A, et al. High titers of neutralizing SARS-CoV-2 antibodies six months after symptom onset are associated with increased severity in COVID-19 hospitalized patients. Virology Journal. 2023;20(1).	Unvaccinated population
Siedner MJ, Boucau J, Gilbert RF, Uddin R, Luu J, Haneuse S, et al. Duration of viral shedding and culture positivity with postvaccination SARSCoV-2 delta variant infections. JCI Insight. 2022;7(2).	More complete article included of the same study
Singh AK, Stellrecht KA, Arunachalam T, Barman TK, Robek MD, Waxman MJ, et al. Lack of active SARS-CoV-2 virus in a subset of PCR-positive COVID-19 congregate care patients. Journal of Clinical Virology. 2021;141.	Study period not reported (probably pre-vaccination period based on publication date and follow-up)
Takahashi K, Ishikane M, Ujiie M, Iwamoto N, Okumura N, Sato T, et al. Duration of Infectious Virus Shedding by SARS-CoV-2 Omicron Variant–Infected Vaccinees. Emerging Infectious Diseases. 2022;28(5):998-1001.	No serial samples within individuals
Van Kampen JJA, van de Vijver DAMC, Fraaij PLA, Haagmans BL, Lamers MM, Okba N, et al. Duration and key determinants of infectious virus shedding in hospitalized patients with coronavirus disease-2019 (COVID-19). Nature Communications. 2021;12(1).	Study conducted during pre-vaccination period

Table S3. Study characteristics of the included studies

Reference	Study design	Country	Study period	SARS-CoV-2 variant of concern	Setting/population	Sample size	Age (mean (SD)* or median (IQR)† in years)	Male (%)	COVID-19 vaccination	Vaccination coverage in study population	- Serial sampling viral culture<br>- Definition day 0
Boucau, 2022[1]	Prospective cohort study	USA	July 2021-Jan 2022	Delta<br>Omicron	Non-hospitalized SARS-CoV-2 positive persons	66 (delta 32; omi-cron 34)	delta<br>40 (16)*<br>omicron<br>42 (15)*	delta<br>34%<br>omicron<br>29%	Initial vaccine series or first booster:<br>- Vaccine NR	76%	- 3 times weekly for 2 weeks or until negative RT-PCR test<br>- Diagnosis
Bouton, 2023[2]	Prospective cohort study	USA	Nov 2021-NR	Delta<br>Omicron	SARS-CoV-2 positive university students and employees	92	22 (3)*	38%	Initial vaccine series or first booster:<br>- Ad26.COV2-S<br>- BNT162b2<br>- ChadOx1 nCoV-19<br>- CoronaVac<br>- mRNA-1273	100%	- Daily for at least 10 days<br>- Symptom onset (data extracted) & diagnosis (data not extracted)
Deyoe, 2023[3]	Prospective cohort study	USA	April 2020-Jan 2022	Alpha<br>Delta<br>Omicron	Primary cases in households	70	NR<br>(aged 5+ years)	46%	NR	27%	- Daily for at least 14 days<br>- Symptom onset or diagnosis
Garcia-Knight, 2022[4]	Prospective cohort study	USA	Sept 2020-Oct 2021	Alpha<br>Delta<br>Epsilon	Non-hospitalized SARS-CoV-2 positive persons	84	37.0 (29.0-44.3)†	44%	Initial vaccine series:<br>- Ad26.COV2-S<br>- BNT162b2<br>- mRNA-1273	38%	- Daily for 2 weeks; intermittent days 17, 19, 21, 28<br>- Symptom onset
Hakki, 2022[5]	Prospective cohort study	UK	Sept 2020-March 2021 & May 2021-Oct 2021	pre-Alpha<br>Alpha<br>Delta	Non-hospitalized SARS-CoV-2 positive community contacts	57	41 (29-49)†	40%	2-dose vaccination:<br>- Vaccine NR	44%<br>(54% for participants with data on culture & symptom onset)	- Daily for up to 20 days<br>- Symptom onset
Jang, 2022[6]	Prospective cohort study	South Korea	Dec 2021	Omicron	Mandatorily hospitalized patients	11	38 (33-46)†	45%	Initial vaccine series:<br>- mRNA-1273	18%	- Daily for 2 weeks<br>- Symptom onset

					(i.e. travelers) with mild to moderate COVID-19						
Jung, 2022[7]	Prospective cohort study	South Korea	July-Aug 2021	Delta	Mild SARS-CoV-2 positive persons isolated in a non-hospital community facility	45	37 (25-49)†	69%	2-dose vaccination‡:<br>- Vaccine NR	18%‡	- Daily for 12 days<br>- Symptom onset
Jung, 2023[8]	Prospective cohort study	South Korea	Feb-March 2022	Omicron	Mild or asymptomatic SARS-CoV-2 positive healthcare workers	32	28 (26-33)†	28%	Initial vaccine series or first booster:<br>- BNT162b2<br>- ChAdOx1 nCoV-19<br>- mRNA-1273	100%	- Daily for 9 days<br>- Symptom onset or diagnosis
Kang, 2023[9]	Prospective cohort study	South Korea	Feb 2021-May 2022	Delta Omicron	Hospitalized patients infected with Delta and Omicron variants	82 (delta 48; omi-cron 34)	delta 60 (40-66)†<br>omicron 67 (52-75)†	delta 50%<br>omicron 68%	Initial vaccine series or first booster:<br>- Vaccine NR	49%	- Daily for 15 days<br>- Symptom onset or diagnosis
Ke, 2022[10]	Prospective cohort study	USA	Dec 2020-March 2021	Alpha Gamma	SARS-CoV-2 positive university students and employees	6	Total popu-lation (n=23) 22 (NR)†; range 19-60	NR	Initial vaccine series§:<br>- Ad26.COV2-S<br>- BNT162b2<br>- mRNA-1273	100%§	- Daily for up to 14 days<br>- Diagnosis
Keske, 2023[11]	Prospective cohort study	Turkey	Jan-Feb 2022	Omicron	Non-hospitalized SARS-CoV-2 positive healthcare workers	55	34 (NR)*; range 23-54	22%	≥2 doses vaccination:<br>- BNT162b2<br>- CoronaVac	98%	- Days 5, 7, 10, 14<br>- Symptom onset
Luna-Muschi, 2022[12]	Prospective cohort study	Brazil	Jan 2022	Omicron	Mild SARS-CoV-2 positive healthcare workers	30	29.5 (25-36)†	53%	≥2 doses vaccination:<br>- BNT162b2<br>- ChAdOx1 nCoV-19<br>- CoronaVac<br>- mRNA-1273	100%	- Days 5, 7, 10, 14<br>- Symptom onset
Phuphuakrat, 2022[13]	Prospective cohort study	Thailand	April-Oct 2021	Alpha Delta	Mandatorily hospitalized non-critically ill COVID-19 patients	36	33.5 (27.7-45.6)†	22%	2-dose vaccination:<br>- inactivated vaccine<br>- heterologous inactivated/ChAdOx 1 nCoV-19	39%	- Every other day until negative RT-PCR test<br>- Symptom onset

Salvatore, 2022[14]	Prospective cohort study	USA	July-Aug 2021	Delta	SARS-CoV-2 positive prisoners	93	NR (aged 18+ years)	100%	Initial vaccine series:<br>- Ad26.COV2-S<br>- BNT162b2<br>- mRNA-1273	84%	- Days 0, 3, 5, 7, 9; subset of samples collected on other days<br>- Symptom onset or diagnosis

IQR: interquartile range; NR: not reported; SD: standard deviation; UK: United Kingdom; USA: United States of America; VOC: variant of concern.

* Mean (SD); † Median (IQR); ‡ Data from partially vaccinated (i.e. from 2 weeks after the first vaccination to within 2 weeks after the second vaccination) participants not extracted; § Data from partially vaccinated (i.e. at least 14 days since first dose) and newly vaccinated (i.e. less than 14 days since first dose) participants not extracted.

Table S4. Risk of bias assessment with the Newcastle-Ottawa Scale for cohort studies

Reference	Selection				Comparability	Outcome			Total points	Risk of bias[ǁ]
	Representative-ness of exposed cohort	Selection of non-exposed cohort	Ascertainment of exposure	Demonstration that outcome of interest was not present at start of study	Comparability of cohorts on basis of design/analysis	Assessment of outcome	Follow-up duration	Adequacy of follow up of cohorts		
Boucau, 2022[1]	+	+	+	+	0	+	+	0	6	moderate
Bouton, 2023[2]	0	NA*	+	+	0	+	+	0	4	moderate
Deyoe, 2023[3]	+	+	0	+	0	+	+	0	5	moderate
Garcia-Knight, 2022[4]	+	+	+	+	0	+	+	0	6	moderate
Hakki, 2022[5]	+	+	+	+	0	+	+	0	6	moderate
Jang, 2022[6]	0	+	0	+	0	+	+	0	4	moderate
Jung, 2022[7]	+	+	0	+ ‡	0	+	+	0	5	moderate
Jung, 2023[8]	0	NA*	0	+	0	+	+	0	3	high
Kang, 2023[9]	+	+	0	+	0	+	+	0	5	moderate
Ke, 2022[10]	0	NA[†]	0	+	0	+	+	+	4	moderate
Keske, 2023[11]	0	+	0	+ §	0	+	+	0	4	moderate
Luna-Muschi, 2022[12]	0	NA*	0	+ §	0	+	+	0	3	high
Phuphuakrat, 2022[13]	+	+	0	+ ‡	0	+	+	0	5	moderate
Salvatore, 2022[14]	0	+	+	+	0	+	+	0	5	moderate

NA: not applicable. * 100% vaccinated; † No unvaccinated group; ‡ First culture at day 2; § First culture at day 5; ǁ Low risk of bias: ≥7 points, Moderate risk of bias: 4-6 points, High risk of bias: ≤3 points.

References

1 Boucau J, Marino C, Regan J, Uddin R, Choudhary MC, Flynn JP, et al. Duration of Shedding of Culturable Virus in SARS-CoV-2 Omicron (BA.1) Infection. New England Journal of Medicine. 2022;387:275-7.

2 Bouton TC, Atarere J, Turcinovic J, Seitz S, Sher-Jan C, Gilbert M, et al. Viral Dynamics of Omicron and Delta Severe Acute Respiratory Syndrome Coronavirus 2 (SARS-CoV-2) Variants With Implications for Timing of Release from Isolation: A Longitudinal Cohort Study. Clinical infectious diseases : an official publication of the Infectious Diseases Society of America. 2023;76:e227-e33.

3 Deyoe JE, Kelly JD, Grijalva CG, Bonenfant G, Lu S, Anglin K, et al. Association of culturable-virus detection and household transmission of SARS-CoV-2 - California and Tennessee, 2020-2022. J Infect Dis. 2023.

4 Garcia-Knight M, Anglin K, Tassetto M, Lu S, Zhang A, Goldberg SA, et al. Infectious viral shedding of SARS-CoV-2 Delta following vaccination: A longitudinal cohort study. PLoS Pathogens. 2022;18.

5 Hakki S, Zhou J, Jonnerby J, Singanayagam A, Barnett JL, Madon KJ, et al. Onset and window of SARS-CoV-2 infectiousness and temporal correlation with symptom onset: a prospective, longitudinal, community cohort study. The Lancet Respiratory Medicine. 2022;10:1061-73.

6 Jang YR, Kim JM, Rhee JE, Kim D, Lee NJ, Lee H, et al. Clinical Features and Duration of Viral Shedding in Individuals With SARS-CoV-2 Omicron Variant Infection. Open Forum Infectious Diseases. 2022;9.

7 Jung J, Kim JY, Park H, Park S, Lim JS, Lim SY, et al. Transmission and Infectious SARS-CoV-2 Shedding Kinetics in Vaccinated and Unvaccinated Individuals. JAMA Network Open. 2022.

8 Jung J, Kang SW, Lee S, Park H, Kim JY, Kim SK, et al. Risk of transmission of COVID-19 from healthcare workers returning to work after a 5-day isolation, and kinetics of shedding of viable SARS-CoV-2 variant B.1.1.529 (Omicron). Journal of Hospital Infection. 2023;131:228-33.

9 Kang SW, Kim JY, Park H, Lim SY, Kim J, Chang E, et al. Comparison of secondary attack rate and viable virus shedding between patients with SARS-CoV-2 Delta and Omicron variants: A prospective cohort study. Journal of Medical Virology. 2023;95.

10 Ke R, Martinez PP, Smith RL, Gibson LL, Achenbach CJ, McFall S, et al. Longitudinal Analysis of SARS-CoV-2 Vaccine Breakthrough Infections Reveals Limited Infectious Virus Shedding and Restricted Tissue Distribution. Open Forum Infectious Diseases. 2022;9.

11 Keske Ş, Güney-Esken G, Vatansever C, Beşli Y, Kuloğlu ZE, Nergiz Z, et al. Duration of infectious shedding of SARS-CoV-2 Omicron variant and its relation with symptoms. Clin Microbiol Infect. 2023;29:221-4.

12 Luna-Muschi A, Noguera SV, Borges IC, De Paula AV, Côrtes MF, Larocca C, et al. Characterization of Severe Acute Respiratory Syndrome Coronavirus 2 Omicron Variant Shedding and Predictors of Viral Culture Positivity on Vaccinated Healthcare Workers With Mild Coronavirus Disease 2019. The Journal of infectious diseases. 2022;226:1726-30.

13 Phuphuakrat A, Pasomsub E, Srichatrapimuk S, Kirdlarp S, Suksatu A, Srisaowakarn C, et al. Detectable Duration of Viable SARS-CoV-2, Total and Subgenomic SARS-CoV-2 RNA in Noncritically Ill COVID-19 Patients: a Prospective Cohort Study. Microbiology Spectrum. 2022;10.

14 Salvatore PP, Lee CC, Sleweon S, McCormick DW, Nicolae L, Knipe K, et al. Transmission potential of vaccinated and unvaccinated persons infected with the SARS-CoV-2 Delta variant in a federal prison, July—August 2021. Vaccine. 2022.
